# Research progress of Astaxanthin nano-based drug delivery system: Applications, prospects and challenges?

**DOI:** 10.3389/fphar.2023.1102888

**Published:** 2023-03-09

**Authors:** Siqian Chen, Jiayi Wang, Jiating Feng, Rongrong Xuan

**Affiliations:** ^1^ Department of Obstetrics and Gynecology, The Affiliated Hospital of Medical School, Ningbo University, Ningbo, China; ^2^ School of Medicine, Ningbo University, Ningbo, China

**Keywords:** Astaxanthin, antioxidation, bioavailability, nano-based drug delivery, pharmacology

## Abstract

Astaxanthin (ASX) is a kind of carotenoid widely distributed in nature, which has been shown to extremely strong antioxidative effects and significant preventive and therapeutic effects on cancer, diabetes, cardiovascular disease, etc. However, its application in the medical field is greatly limited due to its poor water solubility, unstable chemical properties and other shortcomings. In recent years, the nano-based drug delivery systems such as nanoparticles, liposomes, nanoemulsions, nanodispersions, and polymer micelles, have been used as Astaxanthin delivery carriers with great potential for clinical applications, which have been proved that they can enhance the stability and efficacy of Astaxanthin and achieve targeted delivery of Astaxanthin. Herein, based on the pharmacological effects of Astaxanthin, we reviewed the characteristics of various drug delivery carriers, which is of great significance for improving the bioavailability of Astaxanthin.

## 1 Introduction

Astaxanthin (ASX) is a fat-soluble xanthophyll carotenoid that is widely distributed in shellfish, crustaceans, and various plants (Xu et al., 2019). Many types of seaweeds can be used for ASX synthesize, such as snow algae, Chlamydomonas, naked algae, and cyanobacteria, among them, Haematococcus pluvialis showed the highest ASX extraction yield (1.5%–3.0%), and is considered as the best biological source of natural ASX (Li et al., 2020). ASX is a powerful quencher of singlet oxygen and a strong scavenger of oxygen free radicals. The molecular structure of ASX contains 13 conjugated polyunsaturated double bonds, result in its antioxidant effect is 10 times greater than lutein, zeaxanthin, canthaxanthin and β-carotene (Guerin et al., 2003; Ambati et al., 2014).

In recent years, ASX has attracted attention because of its remarkable antioxidant properties and significant role in the prevention and treatment of cancer, diabetes, cardiovascular disease, etc., (Guerin et al., 2003). ASX has been approved to be added to food as dietary supplement, colorant and antioxidant, and has become a product with high demand. However, like other carotenoids, ASX has poor aqueous solubility, low absorption rate *in vivo*, and readily degrades upon exposure to either heat, light, or oxygen, thus limiting its application in the field of medicine (Kim et al., 2022).

Nanocarriers are a delivery systems with the advantages of easy surface modification, biocompatibility and targeted drug delivery and release, which can overcome the limitations of traditional drug delivery—from biological distribution to intracellular transport (Mitchell et al., 2021). The combination of ASX and nanotechnology can greatly improve the issues with using natural or chemically synthesized ASX on its own. Encapsulating ASX into a nano carrier can increase its solubility and stability, and further expand its application range. However, the comprehensive comparison of different ASX nano-based drug delivery systems and their applications in the pharmaceutical field have not been systematically discussed. Therefore, this article firstly focuses on the structure of ASX and its antioxidant activity to understand its clinical therapeutic potential. Then we overview the research progress of ASX nano-based delivery system in recent years, and explore the factors that different nanocarriers affect the stability of ASX delivery systems and their favorable effects on ASX bioavailability and controlled release activity, to contribute to the applications of ASX in the clinic.

## 2 Structure and function of ASX

### 2.1 Structure of ASX

**FIGURE 1:**
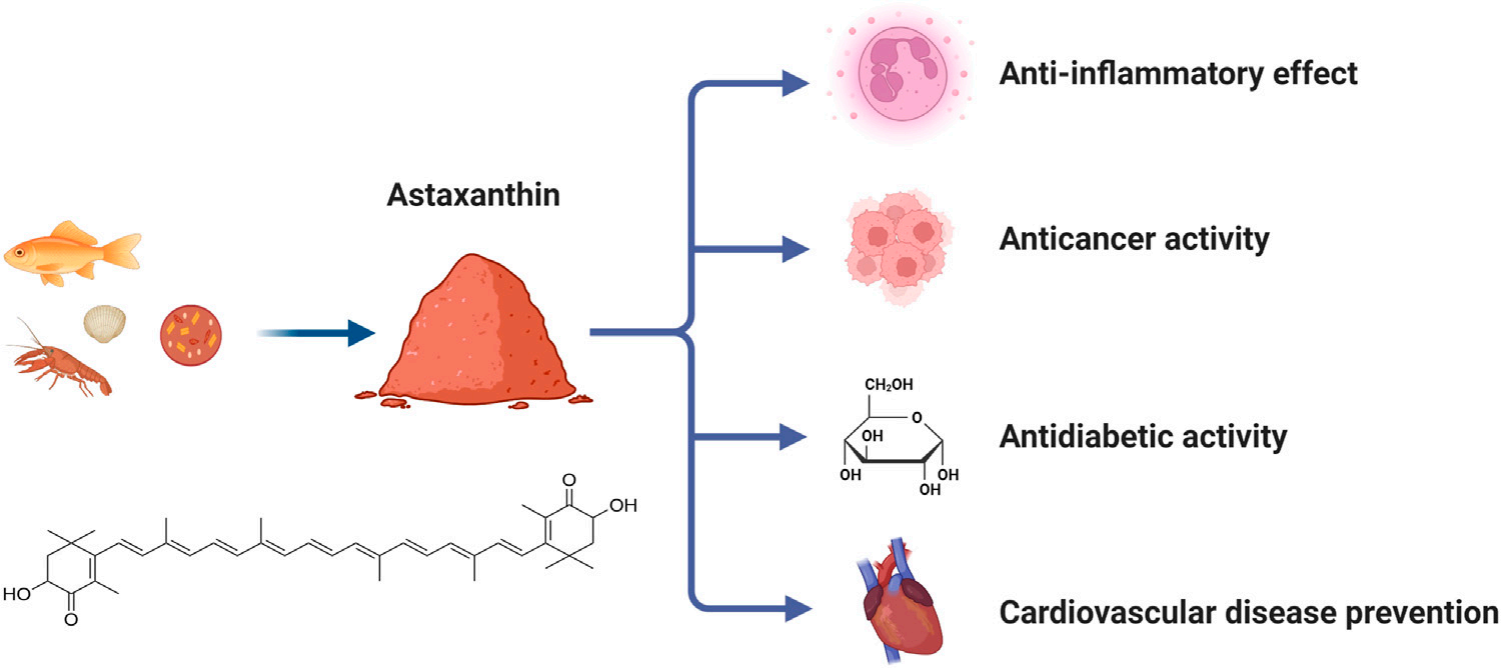
Source, structure and functions of Astaxanthin (ASX).

The chemical formula of ASX is 3,3′-dihydroxy-β, β′-carotene-4,4′-dione (Figure 1) (Zajac et al., 2018). The molecular structure of ASX is composed of four isoprene units in the form of common double bonds, and there are polar ionone rings at either end of the molecule (Chang and Xiong, 2020). The polar–nonpolar–polar structure of ASX enables it to perfectly cross the polar-nonpolar-polar region of cell membrane (Ambati et al., 2014). The ionone rings of ASX can capture free radicals on the outer and inner surfaces of cell membrane, while the conjugated polyene chain can combine with free radicals in the middle of the cell membrane. Here, ASX can convert free radicals into more stable products and terminate a variety of free radical chain reactions, thus owing to its antioxidant capabilities (Fakhri et al., 2018; Mularczyk et al., 2020). However, the high hydrophobicity and poor chemical stability of ASX hinder its antioxidant effects, which also limits its bioavailability. Furthermore, since ASX contains a highly unsaturated molecular structure, it is very sensitive to heat, light, oxides, and acidic or alkaline solutions (Hu et al., 2019).

### 2.2 Antioxidant effects of ASX and its relationship with disease

Depending on the unique molecular structure of ASX, it can neutralize oxygen singlets and scavenge free radicals (Xue et al., 2017). Different from other carotenoids, ASX’s polar structure can bind to cell membrane and reduce lipid peroxidation, which is related to its ability to capture reactive oxygen species (ROS) on both sides of the cell membrane (Snell and Carberry, 2022). In addition, ASX can reduce the formation of ROS by increasing the expression of oxidative stress response enzymes, such as superoxide dismutase (SOD), glutathione peroxidase (GPx) and catalase (CAT) (Hormozi et al., 2019).

A large number of studies have demonstrated that oxidative stress and inflammation are interdependent, which play a role in mediating inflammatory micro-environments. Immune cells at inflammatory sites release a large amount of ROS, resulting in damage of exaggerated tissue and promotion of the pro-inflammatory response (Yin et al., 2021). ROS can induce redox sensitive transcription factors, including nuclear factor kappa B (NF-κB), which is responsible for the production of cytokines, pro-inflammatory chemokines, and adhesion molecules that stimulate phagocytic infiltration. In contrast, ASX exhibits strong antioxidant activity, prevents ROS mediated induction of inflammatory transcription factors, and thereby reduces inflammation (Zarneshan et al., 2020). ASX can also block the gene expression of downstream inflammatory mediators such as IL-1 β, IL-6, and tumor necrosis factor-α (TNF-α) by blocking NF-κB-dependent signaling (Chang and Xiong, 2020). ASX also regulates nuclear factor erythroid 2-related factor 2 (Nrf2), which in conjunction with antioxidant response element (ARE) regulates genes involved in the oxidative stress response to maintain intracellular redox homeostasis (Kohandel et al., 2021). The Nrf2-ARE signaling pathway also increases heme oxygenase-1 (HO-1) expression and inhibits NF-κB to prevent the progression of inflammation (Davinelli et al., 2022). In addition, ASX also participates in p38 MAPK, PI3K/Akt, JAK2/STAT3 and other signaling pathways that endow ASX with antioxidant and anti-inflammatory properties (Yang et al., 2019; Chang and Xiong, 2020; Zarneshan et al., 2020).

ASX has great potential in the prevention and treatment of clinically related diseases due to its antioxidant activity. The abnormal activation of PI3K/Akt signaling pathway is related to the occurrence and development of tumors. ASX can play an anti-cancer role by blocking the transmission of PI3K/AKT, NF-κB and STAT3 signaling paths (Kowshik et al., 2019). Another study found that ASX can reduce the expression of STAT3 at the level of protein and mRNA, thereby inhibiting the proliferation of cancer cells (Sun et al., 2020).

Oxidative stress and inflammation have been known to be key factors driving the progression of diabetes and its related complications. Evidence in the literature supports the idea that ASX can reduce oxidative stress and inflammation through a variety of signaling pathways, such as AMPK, PI3K/Akt/Nrf2, and Nrf2/HO-1 (Chen et al., 2018; Lai et al., 2020; Nishida et al., 2020). ASX has also been shown to protect β-cell function, improve insulin resistance (IR), and increase insulin secretion to reduce blood glucose levels (Gowd et al., 2021).

In addition, ASX increases the bioavailability of nitric oxide (NO) and the activity of antioxidant enzymes through anti-inflammatory and anti-oxidation mechanisms, resulting in decreased platelet activation, vasodilatation, and increased blood flow that maintains hemorheology (Pereira et al., 2021). ASX can also reduce the accumulation of cholesterol in foam cells and the formation of atherosclerotic plaque by increasing the reverse transport of cholesterol by HDL, thus delaying the progression of cardiovascular disease (Zou et al., 2017). The pharmacological functions of ASX are summarized in Figure 1.

## 3 ASX nano-based drug delivery systems

**FIGURE 2:**
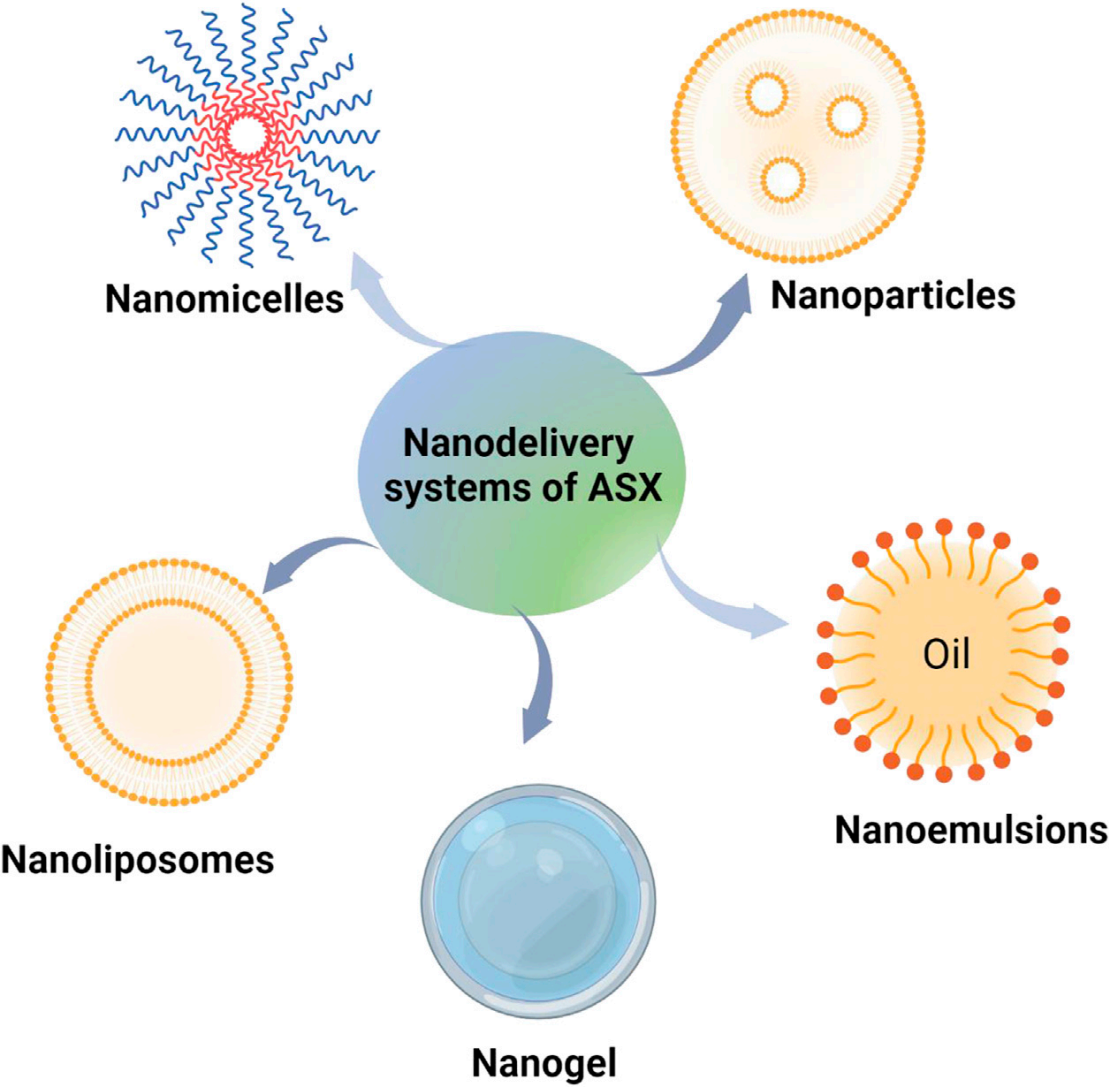
Different ASX nano-based delivery systems.

ASX has the potential to prevent and treat many diseases due to its strong antioxidant activity. However, its clinical application has been hindered by its own defects. The emergence of nano-based drug delivery systems have improved the defects of ASX, such as low water solubility, easy decomposition, low bioavailability, etc. Nano-based drug delivery systems also can improve the clinical application potential of ASX through different embedding methods and carrier selection. At present, the carriers involved in ASX nano-based drug delivery systems mainly include nanoparticles, nanoliposomes, nano emulsions, nanogels and nano micelles (Figure 2). The characteristics and advantages of different ASX nano carriers delivering were shown in Table 1.

**TABLE 1:** Characteristics and therapeutic effects of ASX nano drugs.

Types	Nano carriers	Preparation method	Characterization	Cell/animal models	Advantages	References
Nanoparticles	ASX-Loaded Stealth Lipid Nanoparticles (ASX-SSLN)	Solvent-diffusion technique	The average particle size 130.8±0.2 nm	Human dental pulp stem cells (hDPSC), Primary olfactory ensheathing cells (OECs), Human breASX cancer cells (MCF-7)	Increase antioxidant capacity, bioavailability and maintain drug stability	Pereira et al. (2021)
			The entrapment efficiency > 90%			
	Fe3O4 -Poly (ethylene gly-col) (PEG)-encapsulated ASX nanoparticles (Fe3O4/ATX/Transferrin NPs)	A thermal decomposition method	The average particle size 31±11 nm	C57BL/6 mice, Primary cortical neuron	Higher cell uptake efficiency stronger neuro-protective effect	Su et al. (2021)
			Zeta potential −25.2±6.7 mV			
			The entrapment efficiency 80±6%			
	Ultrasound triggered release nanoparticles (AUT NPs)	Triggered by ultrasound	The average particle size 308.4±51.9 nm	Human stem cells (HL-7702), human embryonic lung fibroblASXs (IMR90), human embryonic kidney cells (HEK-293), human umbilical vein endothelial cells (HUVECs)	Good biological safety and can achieve stable and long-term release in the brain	Qiang et al. (2020)
			Zeta potential −11.2±2.1 mV			
	Chitosan-caseinate-dextran ternary complex (SA-CS/NaCas/Odex) nanoparticles	Across-linking process	The average particle size 119.7 nm	Human hepatic stellate cells (LX-2)	Enhance water dispersion and free radical scavenging ability, and stronger anti hepatic fibrosis effect.	Li et al. (2020)
	Poly (lactic-co-glycolic acid) nanoparticles loaded with ASX (ASX-PLGA NP)	The emulsion solvent evaporation technique	The average particle size 154.4±0.35 nm	Human keratinocytes (HaCaT)	Stronger cell uptake ability and anti-photodamage ability	Chiu et al. (2016)
			Zeta potential 22.07±0.93 mV			
			The entrapment efficiency 96.42±0.73%			
	ASX-loaded triphenylphosphonium bromide modified whey protein isolate -dextran (ASX-loaded TPP-WPI-DX) nano particles	Self-assembly method	The average particle size 193.64 nm	Mouse macrophages (RAW 264.7)	Reduce ROS generation, protect the normal levels of the mitochondrial membrane potential	Sun et al. (2021)
Nanoliposomes	ASX loaded nanostructured lipid carrier (ASXA-NLC)	The technique of melt emulsification—ultrasonic	The average particle size 67.4±2.1 nm	Sprague-Dawley (SD) rats (200 ± 10 g) and Kunming mice (20 ± 5 g)	Better stability and skin permeability	Yang et al. (2019)
			The entrapment efficiency 94.3±0.5%			
	Glucose-PEG600-DSPE ligand modified ASX liposomes (ASX-GLU-LIP)	100 nm polycarbonate membrane extrusion	The average particle size 121.2±3.5 nm	Human mesangial cells (HRMC)	Eliminate excess ROS induced by oxidative stress, superior renal targeted drug delivery	Shen et al. (2018)
			Zeta potential −31.3±0.53 mV			
			The entrapment efficiency, 80.36± 3.26%			
	Liposome encapsulated ASX (LA)	Filtered through a 0.2 µm membrane	The average particle size 240±58 nm	Male SD rats	Improve the bioavailability and completely relieve the acute inflammatory state of rat liver	Yin et al. (2021)
Nanoemulsions	ASX and alpha-tocopherol with sodium caseinate (AS-AT/SC NEs)	Spontaneous and ultrasonication emulsification methods	The average particle size 213.9 nm	Human hepatoma cell line HT-29	Mediate cell apoptosis and exert antitumor effect *in vitro*	Shanmugapriyaet al. (2018)
			Zeta potential −28.09 mV			
	Cellulose nanocrys-tals/nanofibrils loaded ASX nanoemulsion (CNC/CNF@Nes)	Spontaneous emulsification method	The average particle size 206.9 nm	Skin cancer cells L929 and NIH3T3	Regulate signal pathway (PI3K、AKT、ERK、EGFR) and induce apoptosis of cancer cells	Sun et al. (2020)
			Zeta potential −19.34 mV			
	Novel ascorbate palmitate nanoemulsion	Low energy PIT method	The average particle size 20 nm	Porcine sublingual epithelium	Sublingual administration, higher permeability and stability.	Shanmugapriya et al. (2019a)
	ASX and alpha-tocopherol with κ-carrageenan nanoemulsion (AS-TP@KCNE)	Spontaneous and ultrasonication emulsification methods	The average particle size 214.4 nm	human pancreatic cancer cells (PANC1), human epithelial cells (Hela), human colon cancer cells (HT29), mouse wound excision model	Higher biocompatibility and promote wound healing	Xu et al. (2019)
			Zeta potential −28.93 mV			
Nano micelles	Methoxypolyethylene glycol-polycaprolactone polymeric micelles (mPEG-PCL)	Ultrasonication	The average particle size 112.3±16.6 nm	Human bone marrow mesenchymal stem cells	Promote the proliferation of bone marrow mesenchymal stem cells	Zajac et al. (2018)
	ASX micelles self-assembled by hydroxypropylβ-cyclodextrin and glyceryl monostearate	Self-assembled by a mechanochemicamethod	The average particle size 244 nm	SD rats	Improve the ability of scavenging free radicals, strong stability and bioavailability	Nishida et al. (2020)
			Zeta potential −26.7 mV			
Nanohydrogels	ASX hyaluronic acid nanohydrogels (AX / NHS)	Self-assembly	The average particle size 287 nm	Human umbilical vein endothelial cells (HUVECs)	Improve the solubility of ASX in aqueous solution and the ability to neutralize ROS	Pan et al. (2018)
			Zeta potential−45 mV			
	ASX thermoreversible nasal gel (ATX-NLC in situ gel)	Melt-emulsification	The average particle size 225.6±3.04 nm	SD rats	Enhance the transport and release of ASX in the brain	Snell and Carberry, (2022)
			Zeta potential −52.64 mV			
			The entrapment efficiency 65.91±1.22%			

### 3.1 Nanodelivery of drugs to improve aqueous solubility

The water solubility of ASX is a key factor affecting its antioxidant activity and bioavailability. Many coating methods have improved the solubility or dispersion of ASX, such as β-Cyclodextrin complexing, spray-drying and ion gel method, most of which use soluble biopolymers, such as proteins and modified polysaccharides, to specifically interact with ASX (Martinez-Alvarez et al., 2020). Chitosan was coupled with stearic acid and prepared ASX nanoparticles with sodium caseinate and oxidized dextran (SA-CS/NaCas/Odex). Compared with free ASX, encapsulated ASX has greatly improved its water dispersion, significantly enhanced its ability to scavenge free radicals (Hu et al., 2020). Nanodispersions have small particle size and improved water solubility, which can increase the bioavailability of lipophilic components in water-based products. (Shen et al., 2018). The physical and chemical properties of nanodispersions are closely related to emulsifiers. In the ASX nanodispersions prepared from gum Arabic, xanthan gum, pectin and methyl cellulose, the samples produced with gum Arabic showed the minimum average particle size (295 nm) (Anarjan and Tan, 2013b). Polysorbates and sucrose esters of fatty acids are selected as emulsifiers. In the prepared nano dispersions, the particle size of ASX decreases with the increase of the hydrophilicity of the emulsifier and the decrease of the carbon number of the fatty acid in the emulsifier structure, thus showing better dispersion (Anarjan and Tan, 2013a). Nanomicelles can also improve the water solubility of embedded ASX due to the external hydrophilic shell (Cabral and Kataoka, 2014). The ASX micelles self-assembled by a mechanochemical method from hydroxypropyl β-cyclodextrin and glyceryl monostearate had good water solubility, gave them stronger free radical scavenging activity, and improved their bioavailability to be 4 times higher than that of free ASX (Su et al., 2021). Wrapping ASX with hyaluronic acid nano hydrogel can significantly improve the solubility and release in water, and enhance the bioavailability (Montanari et al., 2019).

### 3.2 Nanodelivery of drugs to improve stability

*In vitro* studies, the stability of ASX can be improved by different embedding and packaging technologies. ASX loaded stealth lipid nanoparticles (ASX-SSLNs) prepared by solvent diffusion technology show higher antioxidant capacity than free ASX, which may become a potential carrier for the treatment of Alzheimer’s disease (Santonocito et al., 2021). The ultrasound triggered release nanoparticles (AUT NPs) prepared with perfluorocarbon (PFH), ASX and fluorescent dye IR780 have good stability and can release ASX under ultrasound and pH stimulation, but will not release ASX in advance under non-trigger environment (Cai et al., 2021). In recent years, more and more studies have confirmed that liposome encapsulation can improve the antioxidant activity and cell uptake of ASX. However, due to the thermodynamic instability of liposomes, their integrity is easy to be destroyed, resulting in leakage of packaging materials. A practical way to overcome these problems is to deposit biopolymers (including naturally occurring polysaccharides and proteins) on the surface of liposomes to maintain their structure and increase their mechanical stability (Qiang et al., 2020). The entrapment efficiency is a factor related to the stability. Compared with polysaccharides such as inulin or maltodextrin, surface active biopolymers such as gum Arabic or protein can generally improve the encapsulation efficiency (Martinez-Alvarez et al., 2020). The ASX nanoemulsion prepared with caprylic/capric triglyceride, polysorbate 80 (PS 80) and ascorbyl palmitate as raw materials has an average particle size of 20 nm, an entrapment efficiency of 100% and high stability (Fratter et al., 2019).

### 3.3 Nanodelivery of drugs to improve targeting

Improving the targeted administration of ASX provides new insights for the treatment of clinical diseases. Compared with traditional therapies, using ideal targeted ligands, nanoparticles can help achieve efficient targeted therapy (Chiu et al., 2021). ASX solid lipid nanoparticles have been proved to have unique properties, such as large surface area, high drug loading, small size and wide biological distribution, which can pass through the blood brain barrier, protect the brain from oxidative stress, and achieve the goal of delivering controlled drugs to specific parts (Shanmugapriya et al., 2019a). ASX nanoemulsions play an important role in maintaining ROS generation and mitochondria mediated apoptosis in cancer cells. Through this delivery system, ASX can selectively target mitochondria to achieve specific drug transport and exert anticancer potential (Fakhri et al., 2019). Another glucose-PEG600-DSPE ligand modified ASX liposomes (ASX-GLU-LIP) can specifically transport through the over-expressed GLUT1 on the glomerular mesangial cell membrane to achieve good renal targeted drug delivery (Chen et al., 2020). The nanodispersions formulated with polysorbate 20 can effectively transport ASX to the retina, and reduce loss of photoreceptors and visual damage in mice (Xu et al., 2019).

## 4 Clinical application potential of ASX nano-based delivery systems

Studies have shown that ASX can block the key pathways of neurodegeneration, such as oxidative stress, inflammation and apoptosis. However, the thermal instability and lipophilicity of ASX lead to the failure of its antioxidant effect in many clinical trials, and the lack of delivery systems through the blood brain barrier. ASX nano-pharmaceutics are promising to overcome these limitations and improve drug performance (Fakhri et al., 2019). The ASX thermosreversible nasal gel (ATX-NLC *in situ* gel) prepared by adding poloxamer-127 to ASX nanostructured liposomes can enhance the transport and release of ASX in the brain, and can become an auxiliary treatment drug for Parkinson’s disease (Gautam et al., 2021). ASX nanoparticles (Fe3O4/ATX/Transferrin NPs) coated with transferrin and polyethylene glycol (PEG) have good water dispersibility and biocompatibility. Compared with free ASX, Fe3O4/ATX/Transferrin NPs have a strong neuroprotective effect on oxyhemoglobin induced neuronal damage, which provides new insights for the treatment of subarachnoid hemorrhage (You et al., 2019). ASX nanoparticles also show good stability and higher bioavailability in digestive, blood, skin and other systems (Hu et al., 2019; Hu et al., 2020; Chen et al., 2021).

Nanoemulsions have unique advantages in cancer treatment. ASX and alpha-tocopherol with sodium caseinate nano emulsions (AS-AT/SC-NEs) can exert its anti-tumor potential *in vitro* through mitochondria mediated apoptosis (Shanmugapriya et al., 2019a). ASX nanoemulsions prepared with nanocrystals/nanofibers (CNC/CNF@Nes) combined treatment with low intensity laser can regulate the signal pathway in cancer cells and rely on intracellular signal molecules, such as PI3K, AKT, ERK and EGFR to cause mitochondrial dysfunction, thus inducing apoptosis of cancer cells (Shanmugapriya et al., 2020). ASX and alpha-tocopherol with κ-carrageenan nanoemulsion (AS-TP@KCNE) prepared by spontaneous emulsification and ultrasonic emulsification can significantly recover the weight of mice, reduce the fasting blood glucose level, and improve glucose tolerance through transdermal administration, which has the potential to treat diabetes (Shanmugapriya et al., 2019b).

Nanoliposomes have excellent permeabilities and special targeting characteristics *in vivo* because of their lipid bilayer structure similar to the cell membrane (Pan et al., 2018). ASX loaded nanostructured lipid carrier (ASTA-NLC) prepared by melt emulsification ultrasound technology has a cumulative skin permeability of 174.10 ± 4.38 μg/cm^2^, retention rate within 24 h is 8.00 ± 1.62 μg/cm^2^, indicating that ASTA-NLC has better stability and skin permeability, and can be used as a good transdermal drug delivery route (Geng et al., 2020). Male Sprague Dawley (SD) rats were fed with different doses of liposome encapsulated ASX (LA). Compared with free ASX, LA can completely alleviate the acute inflammatory state of rat liver and can be used as a potential drug release system to treat hepatotoxicity (Gothwal et al., 2015). In addition, nano dispersions and nano micelles, due to their external hydrophilicity, promote the transport of ASX *in vivo*, improve its stability, and show outstanding advantages in drug delivery and targeting therapies (Anarjan and Tan, 2013b; Chiu et al., 2016). ASX nano micelles synthesized with methoxy polyethylene glycol-polycaprolactone (mPEG-PCL) copolymer have the same antioxidant activity, and can promote the significant proliferation of human bone marrow mesenchymal stem cells and differentiate into cartilage, adipogenesis and osteogenesis (Zhang and Peng, 2019).

## 5 Limitations of current application and future development trends of ASX nano-based drug delivery systems

Due to the different materials and preparation processes of various ASX nano-based drug delivery carriers, each delivery systems show some differences, and have different clinical application potential in many systems such as nervous, digestive, endocrine, etc. However, the current ASX nano-based drug delivery systems still have many limitations. ASX nanoparticles have good entrapment efficiency and high stability, but showed poor water solubilities and low loading rate of ASX. Compared with nanoparticles, nanoliposomes have less crystal arrangement in structure and can provide more ASX binding space (Abdol Wahab et al., 2022). But the poor stability and inefficient encapsulation of nanoliposomes together with excessive lipid may cause more serious health problems for obese and hyperlipidemic population (Sun et al., 2021). Nanoemulsions and nanodispersions have good stability, permeability and targeting, but their absorption *in vivo* depends on the type of solvent. The potential toxicity of organic solvent may have adverse effects on human body (Shanmugapriya et al., 2018). So far, the clinical research on ASX nano-based drug delivery systems are very limited, most of which rely on *in vitro* experiments, and there is also a lack of further validation research on the safety of nano materials. The application, efficacy and safety of ASX nano preparation *in vivo* need to be studied more systematically in the future. In recent years, the emergence of various new nano materials has further improved the bioavailability of ASX. The feasibility of application should be considered when selecting an appropriate delivery system. While improving the bioavailability of ASX, the delivery methods should be simplified as much as possible, and oral or skin-based drug delivery methods should be selected to promote the application of ASX in prevention, treatment and healthcare.

## 6 Conclusion

In conclusion, ASX has strong antioxidant activity, and has many beneficial effects including anti-inflammatory properties, anti-cancer effects, blood glucose regulation, and anti-cardiovascular disease properties. However, due to its low bioavailability and instability, it lacks further clinical practice. Using novel nanotechnologies to encapsulate ASX can significantly enhance the water solubility, stability, targeting, and bioavailability of ASX to enhance its pharmacological effects. Different nano carriers can provide new opportunities and methods for ASX encapsulation for disease prevention and treatment. In recent studies, the combination of organic nanoparticles and ASX has gained attention, however research at this stage is mainly limited to preclinical assessments. Compared with free ASX, nano-based drug delivery systems have achieved remarkable results with different materials, thereby vastly improving delivery efficiency. However, the efficacy of ASX nano-based delivery systems have not been tested clinical, thus requiring further research. In the future, the identification and development of novel nano carriers should be explored to improve the efficacy of ASX in preclinical and clinical studies.
